# Using Magneto-Inertial Measurement Units to Pervasively Measure Hip Joint Motion during Sports

**DOI:** 10.3390/s20174970

**Published:** 2020-09-02

**Authors:** Rachel E. Horenstein, Yohann R. Goudeau, Cara L. Lewis, Sandra J. Shefelbine

**Affiliations:** 1 Department of Mechanical & Industrial Engineering, Northeastern University, Boston, MA 02115, USA; horenstein.r@northeastern.edu (R.E.H.); yohann.goudeau@hotmail.fr (Y.R.G.); 2 Department of Physical Therapy & Athletic Training, Boston University, Boston, MA 02215, USA; lewisc@bu.edu; 3 Department of Bioengineering, Northeastern University, Boston, MA 02115, USA

**Keywords:** pervasive measurements, hip joint motion, wireless sensor motion-capture system, magneto-inertial measurement units, cam morphology

## Abstract

The use of wireless sensors to measure motion in non-laboratory settings continues to grow in popularity. Thus far, most validated systems have been applied to measurements in controlled settings and/or for prescribed motions. The aim of this study was to characterize adolescent hip joint motion of elite-level athletes (soccer players) during practice and recreationally active peers (controls) in after-school activities using a magneto-inertial measurement unit (MIMU) system. Opal wireless sensors (APDM Inc., Portland OR, USA) were placed at the sacrum and laterally on each thigh (three sensors total). Hip joint motion was characterized by hip acceleration and hip orientation for one hour of activity on a sports field. Our methods and analysis techniques can be applied to other joints and activities. We also provide recommendations in order to guide future work using MIMUs to pervasively assess joint motions of clinical relevance.

## 1. Introduction

Researchers, clinicians and athletes are continuing to increase their use of wireless sensors to measure human movement. Portable, low-cost wireless sensors are advantageous because they can monitor motion in non-laboratory settings over long durations of time. Wireless sensor systems have been validated and used to measure motion for a variety of joints [1,2,3,4,5,6,7,8,9,10,11] with procedures that become increasingly complicated with increasing degrees of freedom [4,10,11]. Inertial measurement units (IMUs) and magneto-inertial measurement units (MIMUs) are arguably the two most popular types of wireless sensors for measuring motion. Both measure acceleration and angular velocity. MIMUs additionally measure the magnetic field in the surrounding environment.

There is a strong focus on using wireless sensors to monitor athletes [12,13,14,15,16] with emphasis on performance assessment [9,12,17,18,19,20] and injury prevention [21,22]. To evaluate player performance, single IMU acceleration-derived measures have been adopted to analyze intensity throughout the course of sports practices. The instantaneous rate of change of tri-axial acceleration, for example, has been used to quantify “external training loads” [19,23]. Often, studies use only one wireless sensor, typically secured to an individual’s torso [13,14,19,23]. This approach, however, eliminates the ability to analyze joint motion, which requires at least two sensors in order to relate the orientation of two neighboring body segments. 

Joint kinematics of both upper [24,25] and lower [10,15,26,27,28,29] extremities have been measured in various athletic populations using (M)IMUs. However, these studies come with limitations since joint angles were often measured in controlled settings for short durations of time (on the order of several minutes) with assumptions that are not generalizable to natural environments. Joint-specific anatomical constraints are often applied to correction algorithms over the duration of data collection, such as assuming the elbow is a hinge joint constantly restricted to 0° adduction/abduction [30] or imposing known constraints of a specific joint’s range of motion [2]. For example, hip joint angles have been measured during stationary cycling with the assumption of planar hip joint motion [31]. While this planar assumption is suitable for joint motions that occur predominantly in a single plane (such as hip motion during walking or cycling), it is not appropriate for all movements/tasks.

While studies in motion capture laboratories have well-documented hip motion over the course of a single step, we have little knowledge of what the hip does over the course of an hour. Statistical analysis of time series data may be relevant to and critical for understanding variations in bone shape. Cam morphology, for example, is characterized by abnormal convexity at the anterosuperior portion of the femoral head-neck junction. It is highly prevalent (>50%) in athletes who participated in elite-level soccer [32,33,34,35,36,37,38], basketball [39,40] and ice hockey [41,42,43,44,45] during adolescence and is thought to be the result of a mechanoadaptive response to altered hip joint mechanics associated with these at-risk sports [32,33,34,36,39,40,41,42,43,45,46,47,48,49,50,51,52,53]. However, the altered stresses suspected to contribute to cam morphology have not yet been quantified. Cam morphology may be linked to the intensity of hip joint motion or to the hip joint orientation of at-risk athletes. Answering this question requires knowledge of the magnitude and direction of joint motions and the lack of research in this area is likely due to the inherent challenges with quantifying these measures in a non-laboratory setting. Joint motion and forces are traditionally assessed in a laboratory with concurrent use of a camera-based motion capture system and force plates. Access to these technologies is unfortunately unfeasible in sporting arenas, mainly due to lack of portability and small capture volume. While wireless sensors cannot determine the magnitude of loading, they do provide insight to hip joint orientation and, by extension, provide indication of loading direction.

The aim of this study was to develop methods for analyzing hip joint motion captured with MIMUs in a natural environment (pervasively). We applied our methods to a comparison of the hip joint motion during sports practices associated with high risk for cam morphology (soccer players) and during recreational afterschool activities (controls). Our comparison methods provide measures of motion intensity and orientation.

## 2. Materials and Methods

### 2.1. Participants

Healthy male elite-level athletes (soccer players) and recreationally active peers (controls) with no history of hip injury were recruited to participate. Participant demographics are shown in Table 1. All adolescents were between 8–12 years old to ensure a skeletally immature cohort with open proximal femoral growth plates. Participant assent and legal guardian consent were obtained prior to study procedures. This study received ethics approval from the Northeastern University Institutional Review Board (IRB# 14-11-21). 

### 2.2. Data Collection Methods

**Figure 1:**
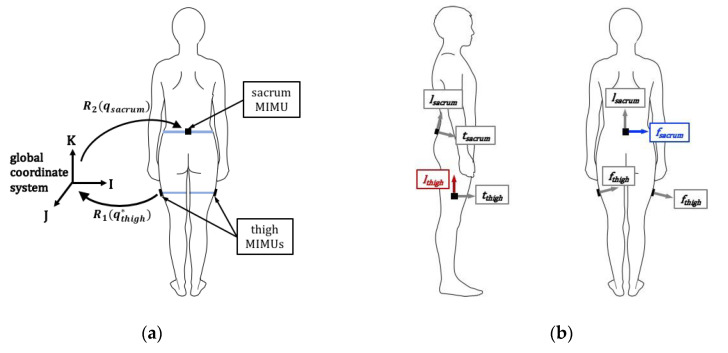
(**a**) Three magneto-inertial measurement units (MIMUs) were used to create a sensor-based motion capture system. MIMUs were placed at the sacrum and laterally on each thigh and were secured with elastic straps (shown in light blue) and Cover Roll Stretch^®^ (not shown). The conjugate of the normalized thigh quaternion found in Motion Studio was used to rotate the thigh sensor coordinate system (3 × 3 identity matrix) to the global coordinate system. The thigh orientation in the global coordinate system was then rotated to the sacrum sensor coordinate system using the sacrum sensor quaternion. (**b**) Hip joint coordinate systems were defined using sensor coordinate systems. A functional calibration procedure was used to better align these definitions with anatomically relevant ones.

Adolescent hip joint motion during sports practices (athletes) and typical recreational activities (controls) was measured using a system of three MIMUs (Opal wireless sensors v1, APDM Inc., Portland, OR, USA). MIMUs were positioned directly on the participant’s skin at the sacrum and laterally on each thigh and were secured with elastic bands and Cover Roll Stretch^®^ to limit displacement (Figure 1a). MIMUs were configured for synchronized logging at 128 Hz. Each participant practiced his sport (or carried out his regular after-school activities for controls) while data were recorded to an on-board flash memory. Prior to the start of activity, participants were instructed to assume a neutral standing posture for 5 s and then walk for at least 10 s. These data were used for the functional calibration procedure (Section 2.5) during data post-processing. The MIMUs placed on two control participants moved during data collection and these data were excluded from analysis. While soft tissue artifact is a recognized source of error in wireless sensor recordings [10,50], it is also a known source of error in camera-based motion capture systems. We previously validated the methodology we used for wireless sensor data collection in a camera motion capture laboratory [11]. We found that the mean absolute difference between hip joint flexion/extension and adduction/abduction calculated with these systems was mostly <6°. The exception was for motion characterized by hip flexion exceeding 90°, where the mean absolute difference of adduction/abduction was slightly greater (7.2°).

Following data collection, data were imported to Motion Studio Software (APDM Inc., Portland, OR, USA). We used the quaternions (also commonly referred to as quaternion estimates or orientation estimates) calculated with this software to define the orientation of each sensor (thighs and sacrum) in the global Earth inertial reference frame. The Motion Studio algorithm estimates quaternions with a custom state space model and causal Kalman filter. The variable weight magnetometer model (software default) places a varying weight on the magnetometer measurement, which accounts for deviations in the inclination angle (accelerometer) and magnetic field magnitude (magnetometer) relative to values obtained during the sensor hardware calibration. Sensor data (including APDM Inc. derived quaternions) were exported to MATLAB (Mathworks Inc., Natick, MA, USA) for further post-processing. 

### 2.3. Coordinate System Definitions

There are multiple coordinate systems that should be understood in the contexts of measuring human movement and relating the orientations of two or more sensors. Their definitions are included below for clarity.

- Sensor coordinate systems are non-changing, orthonormal, local reference frames that are embedded in each wireless sensor. The three orthonormal axes (i_local_, j_local_, k_local_) that define the coordinate system lie parallel to the rectangular casing of the sensor with the origin located at the casing’s center. Each sensor measures and records data relative to its own local coordinate system.
- The global coordinate system is a non-changing, orthonormal, inertial reference frame that is common for all sensors. The three orthonormal axes (I_global_, J_global_, K_global_) are defined by the directions of the Earth’s magnetic north (I_global_) and gravity (K_global_). Data recorded in a sensor coordinate system data can be mapped to the global coordinate system using quaternion operators.
- Body segment coordinate systems are non-changing, orthonormal, anatomically relevant, local coordinate systems that are specific to a given body segment and are defined by body-fixed axes. These coordinate systems are also commonly referred to as “anatomical segment frames” in the literature. The orthonormal body-fixed axes (L-axis, F-axis, T-axis) are defined in accordance with the specific anatomy of the body segment [54,55]. The l (longitudinal) axis coincides with the body segment’s longitudinal axis and is inferosuperiorly oriented. The f (flexion) axis is mediolaterally orientated, coinciding with the body segment axis about which flexion/extension occurs. The t (third) axis is the third body-fixed axis defined by the cross-product l × f. When using camera-based motion capture systems, these axes are “embedded” in body segments by incorporating positional data of numerous anatomical markers. However, for sensor-based systems, a single sensor on a body segment does not provide sufficient anatomical information to embed the coordinate system in anatomical based coordinates and therefore does not directly correspond with International Society of Biomechanics (ISB) recommended segment frames.
- Joint coordinate systems are changing, joint specific coordinate systems that are defined by the relative orientation between two neighboring body segments. The unit vectors that define these coordinate systems (denoted by e_1_, e_2_, e_3_) are defined by the body-fixed axes of the two neighboring segments. Joint coordinate systems are not necessarily orthogonal.

This study focused on the hip joint, the coordinate system of which is defined by the body-fixed axes of the pelvis and thigh body segments. The thigh and pelvis body-fixed axes were defined using the thigh and sacrum sensor coordinate systems (Figure 1b). To maintain consistency across body segments, the positive L-Axis was chosen to coincide with the inferior direction, the positive T-Axis the anterior direction and the positive F-Axis by direction of t × l for all three body segments.

The hip joint coordinate system unit vectors were defined as follows [54,55]:

(1) $$e_{1}\;=\;f_{\mathrm{sacrum}}$$

e_3_ = l_thigh_ (2)

(3a) $$e_{2}\;=\left(\frac{e_{3}\;\times \;e_{1}}{\left|e_{3}\;\times \;e_{1}\right|}\right)*\;A,$$

(3b) $$\mathrm{where}\;A\;=\left\{\begin{array}{c} -1\;\mathrm{if}\;\left(\left(e_{3}\;\times \;e_{1}\right)\;\cdot \;t_{\mathrm{thigh}}\;<\;0\right)\;\mathrm{and}\;\left(\left((e_{3}\;\times \;e_{1}\right)\;\times \;e_{3})\times \;f_{\mathrm{thigh}}\;>\;0\right)\\ 1\;\mathrm{otherwise} \end{array}\right\}$$

Hip joint angles can be calculated with these anatomical axes according to previous methods [11,54,55]:

Flexion/extension (angle of rotation about the e_1_ axis):

θ*_FE_* = cos^−1^(e_2_ · t_sacrum_) ∗ sign(e_2_ · l_sacrum_). (4)

Adduction/abduction (angle of rotation about the floating e_2_ axis):

(5) $$\theta_{AA}\;=\;\cos^{-1}(r\;\cdot \;l_{\mathrm{thigh}})\;*\;\mathrm{sign}(f_{\mathrm{sacrum}}\;\cdot \;l_{\mathrm{thigh}}),\;\mathrm{where}\;r\;=\;\frac{f_{\mathrm{sacrum}}\;\times \;e_{2}}{\left|f_{\mathrm{sacrum}}\;\times \;e_{2}\right|}.$$

Internal/external rotation (angle of rotation about the e_3_ axis)

θ*_IE_* = cos^−1^(e_2_ · t_thigh_) ∗ sign(e_2_ · f_thigh_), (6)

where * indicates scalar multiplication. The sign terms in Equations (4)–(6) are scaling factors of +1 (if dot product > 0) or −1 (if dot product < 0). These sign terms ensure that clockwise rotations about e_1_, e_2_ and e_3_ are positive and therefore consistent with the commonly accepted direction of positive angles in a right-handed coordinate system. 

### 2.4. Coordinate System Transformations

An Opal MIMU records acceleration, angular velocity and magnetic field data in its own sensor coordinate system. Since each sensor coordinate system is local to the sensor (and therefore independent), data from two different sensors cannot be related without a known forward and inverse transformation to the common global coordinate system. We used two rotation matrices (R_1_ and R_2_ shown in Figure 1a) to find the relative orientation between the thigh and sacrum sensors. Both R_1_ and R_2_ were calculated directly from the quaternions obtained from the Motion Studio Software. The first rotation matrix, R_1_, was found in MATLAB by inputting the normalized conjugate of the thigh quaternion given by Motion Studio into the built-in function *quatrotate*. R_1_ rotated the orientation of the thigh sensor coordinate system (i_local,thigh_ = [1 0 0], j_local,thigh_ = [0 1 0], k_local,thigh_ = [0 0 1]) to the global coordinate system (defined by I_global_, J_global_, K_global_). The second rotation matrix, R_2_, was found in MATLAB by inputting the normalized sacrum quaternion given by Motion Studio into *quatrotate*. R_2_ rotated the orientation of the thigh sensor coordinate system in global coordinate system to the sacrum sensor coordinate system (defined by i_local,sacrum_ = [1 0 0], j_local,sacrum_ = [0 1 0], k_local, sacrum_ = [0 0 1]). These two successive rotation matrices, R_1_ and R_2_, were applied to the thigh sensor coordinate system (i_local,thigh_ = [1 0 0], j_local,thigh_ = [0 1 0], k_local,thigh_ = [0 0 1]) using the MATLAB Aerospace Toolbox (Mathworks, Natick MA, USA). The thigh tri-axial acceleration data were transformed to the sacrum sensor coordinate system using these same quaternion operators and methods.

For both the acceleration data and orientation data, we analyzed data in the sacrum sensor coordinate system. Analyzing the data in the global coordinate system would have yielded the same results. We chose to analyze data in the sacrum sensor coordinate system because visualizing the motion of the thigh from the reference frame of the pelvis was the most straightforward way to express the data.

### 2.5. Functional Calibration 

**Figure 2:**
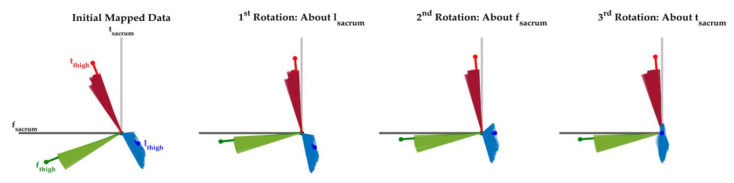
An example of the functional calibration procedure used to define anatomically relevant body fixed axes. Three successive rotations were used. This figure shows these rotations from the viewpoint of l_sacrum_, where +t_sacrum_ points anteriorly and +f_sacrum_ points to the participant’s right-hand side. In this example, the flexion/extension and adduction/abduction hip joint angles during neutral standing are 0° and 0.21° respectively.

After determining the orientation of the thigh sensor coordinate system in the sacrum sensor coordinate system, we used a functional calibration procedure to reorient the sacrum sensor coordinate system to an anatomically appropriate hip joint coordinate system. This functional calibration procedure used two assumptions [11]. The first was that hip motion during walking occurs in the sagittal plane. Using this assumption, a principal component analysis (PCA) was used to determine the direction perpendicular to the sagittal plane (motion of the thigh during walking). We applied PCA to the orientation of the longitudinal thigh sensor axis (l_thigh_) in the sacrum sensor (refer to Figure 1b for initial definitions of sensor body-fixed axes). The anatomically relevant mediolateral axis was defined by the principal direction with smallest eigenvalue. The first rotation, which occurred about l_sacrum_, aligned the identified mediolateral axis (found with PCA analysis of data during walking) with f_sacrum._ The second assumption was that neutral standing corresponds to 0° hip flexion/extension and adduction/adduction. This second assumption requires that l_sacrum_ and l_thigh_ be aligned during neutral standing. While keeping the newly aligned mediolateral axis invariant, a second rotation was applied about f_sacrum_ so that no component of l_thigh_ was contained in the direction of t_sacrum_ during neutral standing. This rotation ensured 0° flexion/extension during neutral standing. Lastly, a third rotation matrix was applied about t_sacrum_ so that no component of l_thigh_ was contained in the direction of f_sacrum_ during neutral standing. This rotation ensured 0° abduction/adduction during neutral standing. A demonstration of these three successive rotations can be found in Figure 2. After transforming the thigh sensor data to the sacrum sensor coordinate system and functionally calibrating the sacrum sensor coordinate system, we were able to proceed with analyzing motion intensity and hip joint orientation data.

While some calibration procedures do not require movement [56], the calibration data can be difficult to correctly identify in a long duration (1 + h) continuous sensor recording. Dynamic hip joint functional calibration procedures that are applied to reorient the sensor frame to the anatomical frame typically require more than one movement. The functional calibration procedure proposed by Fasel et al. [10], for example, requires three movements in addition to upright neutral standing. We found that reducing the set-up time was an important consideration for our adolescent athletic participants. Therefore, we chose a functional calibration procedure that minimized the necessary tasks (walking and neutral standing) before practices.

### 2.6. Motion Intensity

**Figure 3:**
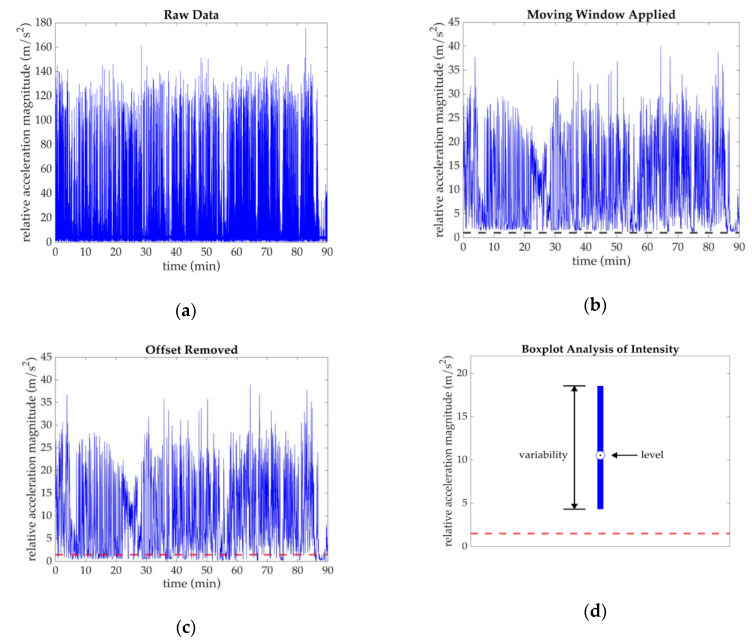
Example of data post-processing methods for one athlete. (**a**) Raw relative acceleration magnitude data between the thigh and sacrum sensors; (**b**) Data after application of 3 s average moving window. A baseline offset (black dashed line) was determined by the minimum value of the data after the application of the average moving window; (**c**) The baseline offset was subtracted from the moving window applied data set to find the intensity. The red dashed line indicates the 1.5 m/s^2^ dynamic threshold; (**d**) Intensity data above the dynamic threshold (red dashed line) for a duration of ≥2 s were condensed into a boxplot to highlight the intensity “level” (50th percentile) and “variability” (range between the 20–80th percentiles). In this example, the boxplot contains 565,970 data points, which is equivalent to ≈74 min of motion data.

To determine intensity, we first calculated the magnitude of the relative acceleration between the thigh and sacrum sensors (Figure 3a). The relative acceleration ([$a_{f\_relative}\;a_{l\_relative}\;a_{t\_relative}])$ was found as the difference between the sacrum acceleration and the thigh acceleration measured in the sacrum sensor coordinate system (i.e., mapped thigh acceleration data):

(7a) $$a_{f\_relative}\;=\;a_{f\_sacrum}-a_{f\_thigh}$$

(7b) $$a_{l\_relative}\;=\;a_{l\_sacrum}-a_{l\_thigh}$$

(7c) $$a_{t\_relative}\;=\;a_{t\_sacrum}-a_{t\_thigh}.$$

The magnitude of the relative acceleration was data determined as:

(8) $$a_{mag}\;=\;\sqrt{{\left(a_{f\_relative}\right)}^{2}+{\left(a_{l\_relative}\right)}^{2}+{\left(a_{t\_relative}\right)}^{2}}.$$

A 3 s average moving window was applied to the magnitude of the relative acceleration data (Figure 3b). This window duration was chosen in order to remove outliers (i.e., act as a filter) while also ensuring that characteristics of the data were not “lost” by an inappropriately large window size. Based on in-field observations during sports practices, a 3 s window was shorter in duration than the shortest bursts of activities and therefore deemed appropriate. Because we were interested in overall activity, not peak activity, for this study, a 3 s moving average window was appropriate. A shorter window would be necessary if peak accelerations were of interest. After applying the average moving window, baseline magnitudes during standing were greater than 0 m/s^2^ (Table 2). These offsets were likely mostly due to participants never being fully stationary and the outcome effects of a moving average window. Based on in-field observations, adolescents shift their stances during huddles or standing, a motion that lends itself to small differences in pelvis and thigh body segment motion (captured by the sacrum and thigh sensors respectively) and varying soft tissue artifact. With the application of an average moving window, larger changes in acceleration during brief movements would affect the 3 s average. To correct for these baseline offsets, we subtracted each participant’s minimum baseline value from their magnitude data (Figure 3c). Although not an ideal data correction approach, this method was deemed adequate given that each participant’s offset was constant throughout the practice. A two-tailed unpaired t-test confirmed no significant differences between athletes’ and controls’ offsets, indicating no cohort dependency. In the work presented here, “intensity” data refers to the relative acceleration magnitude data after application of the 3 s moving window and the offset correction.

Intensity data were analyzed during dynamic periods, which were defined by intensity data exceeding a dynamic threshold (1.5 m/s^2^) for a duration of ≥2 s. We chose a dynamic threshold of 1.5 m/s^2^ for a duration of ≥2 s based on careful observation and observer notes of activities and drills during practice. The 1.5 m/s^2^ threshold was similar to our baseline offset values and the ≥2 s duration ensured capture of drills and play (i.e., periods of continuous movement and not discrete time points). We chose to only analyze intensity data above the dynamic threshold because joint forces are likely highest during these periods and most important in understanding the mechanism of cam morphology. 

To gain an understanding of the overall characteristics of motion intensity throughout a practice, intensity data exceeding the dynamic threshold for ≥2 s were condensed in a boxplot. As illustrated in Figure 3d, motion intensity was quantified with a level (defined by the 50th percentile) and variability (defined by the range between the 20–80th percentiles). Differences in level and variability between athletes and controls were compared using a two-tailed, unpaired t-test (0.05 significance level).

### 2.7. Joint Orientation

**Figure 4:**
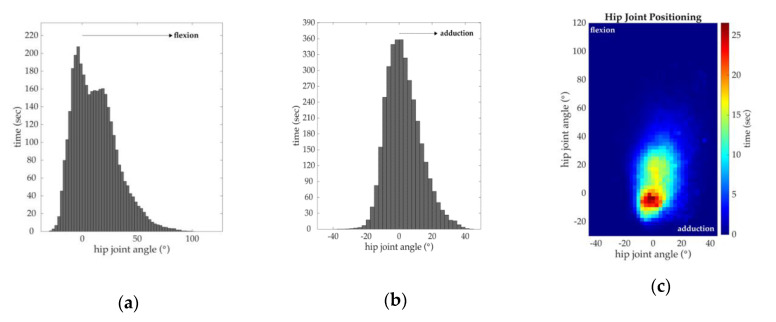
Example of methods applied to a soccer athlete measuring hip motion during a soccer practice. (**a**) Histogram highlighting the most frequent hip joint flexion/extension angle; (**b**) Histogram highlighting the most frequent hip joint adduction/abduction angle; (**c**) Spatial histogram highlighting the most frequent hip joint orientation (simultaneously describes flexion/extension and adduction/abduction).

To gain an understanding of the overall characteristics of hip joint orientation throughout a practice, we plotted the flexion/extension and adduction/abduction hip joint angles on spatial histograms during periods corresponding to those used in the intensity data analysis (intensity > 1.5 m/s^2^ for a duration of ≥2 s). Figure 4 shows two histograms during an athlete’s sports practice, which separately indicate the most frequent hip joint flexion/extension and adduction/abduction angles. The disadvantage of using separate histograms for each joint angle is that they only show data in one anatomical plane and do not show the most frequent hip joint orientation (simultaneous quantification of more than one joint angle). To address this limitation, we created spatial histograms that incorporate data measured in two orthogonal anatomical planes and highlight these data frequencies. We chose not to analyze hip joint internal/external rotation angles because of their strong dependence on the surrounding magnetic field [11]. Video S1 (supplementary material) demonstrates different viewpoints of hip joint motion.

Hip joint orientation data within localized regions of spatial histograms are not independent from data in neighboring spatial regions and are therefore an example of continuous spatial data. Comparison of spatially continuous data between groups requires two-dimensional statistical parametric mapping (spm2d). This technique uses random field theory to conduct statistical tests on data over a continuous domain and addresses the multiple comparison problem introduced by non-independent neighboring data [57,58]. Two-tailed, two-sample unpaired t-tests were used to test the null hypothesis that the hip joint orientation data of groups were equal. This test was performed within the bounds of anatomically feasible concurrent hip joint flexion/extension and adduction/abduction [59]. Non-parametric statistical inference on hip orientation data was performed to detect differences between athletes and controls (0.05 significance level) using the spm1d toolbox developed by Todd Pataky (available for download from http://spm1d.org).

## 3. Results

### 3.1. Motion Intensity

**Figure 5:**
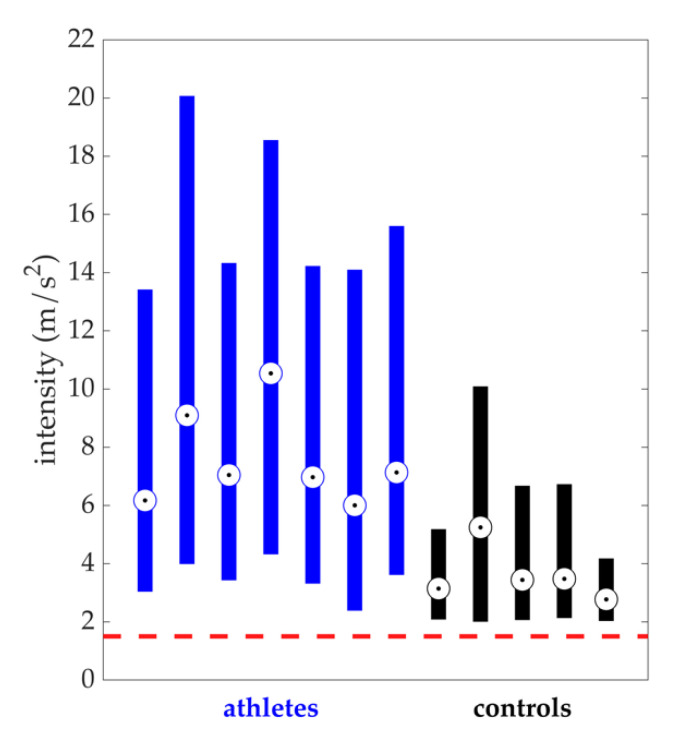
Athlete individual intensity data are shown in blue and control individual intensity data are shown in black. Data from one hip were analyzed for each player. The horizontal dashed line (red) corresponds to the dynamic threshold value (1.5 m/s^2^). The means of each group are shown in Table 3.

The individual motion intensity data of athletes and controls are shown in Figure 5. The mean intensity level and variability of each group are shown in Table 3. Athletes displayed a greater intensity level and variability than controls (*p* < 0.001). They also had ≈ 4× times as many minutes over the dynamic threshold.

### 3.2. Joint Orientation

**Figure 6:**
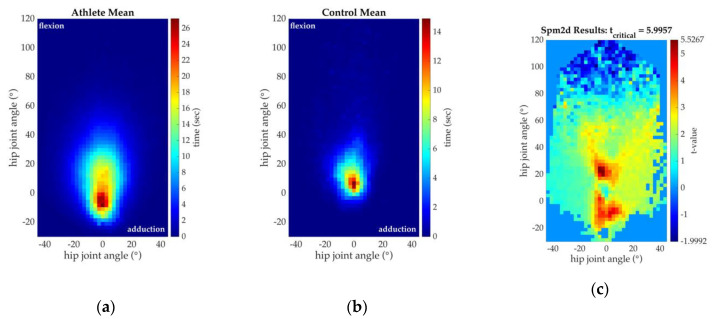
Spatial histograms of hip joint orientation. (**a**) Mean hip joint orientation of all athletes (soccer players) during sports practices. The colormap indicates whether a given orientation occurs with a high (red) or low (blue) frequency; (**b**) Mean hip joint orientation of all controls during typical recreational activities. The colormap indicates whether a given orientation occurs with a high (red) or low (blue) frequency; (**c**) Spm2d t-value test result at each spatial location of hip joint orientation. The colormap indicates the t-test value determined at a given orientation. No significant differences between athletes and controls (t_critical_ = 5.9957) were detected.

Figure 6 shows the average hip joint orientation of athletes and controls and the corresponding raw spm2d t-value test result. The maximum t-value reached (5.5267) was less than the adjusted t_critical_ (5.9957); therefore, no significant difference in hip joint orientation between groups was detected (*p* > 0.05). It is important to note that our sample size (*n* = 7 athletes, *n* = 5 controls) was small and likely underpowered.

## 4. Discussion

Using wireless sensors to measure joint motion eliminates the need to capture data within the confines of a laboratory space and allows real-time motion capture during a range of activities. With the ability to measure multiple kinematic variables (acceleration and angular velocity) and to relate these variables across sensors (quaternion estimates), sensor technologies can be used to determine overall characteristics of motion and can be applied to clinically relevant movement analysis. 

### 4.1. Novel Methods for Measuring Hip Motion in the Natural Environment

We developed a new approach for analyzing hip joint motion collected with wireless sensors. We used two measures to analyze motion—intensity (acceleration-derived measure) and hip joint orientation (orientation-derived measure). We applied our methods to measure hip joint motion of athletes (elite-level adolescent soccer players at risk for cam morphology) and controls (recreationally active peers) in an effort to gain initial insight into differences between these groups. We compared differences in intensity (level, variability) using t-tests and differences in joint orientation (spatial histogram) with spm2d analysis. 

To date, analysis of joint angles using wireless sensors has typically been performed by analyzing joint angles separately. For example, Kirking et al. analyzed upper arm motion during daily living activities over the course of 8 h with separate histograms and percentiles of flexion/extension, abduction/adduction and internal/external rotation joint angles [2]. Spm2d is a well-established robust method that can be applied to comparing 2d spatial distributions of joint orientation frequency. Our presented application of spm2d provides researchers and clinicians with a new approach for analyzing hip joint orientation (i.e., flexion/extension in combination with adduction/abduction) as a continuous variable over a surface. By removing the time series component of the data, this method allows us to analyze motion characteristics while accounting for neighboring spatial dependency. In the current study, the amount of time spent in dynamic periods by athletes was significantly greater than the time of controls (mean ± SD: 69.76 ± 7.69 min and 16.85 ± 9.16 min, respectively) (*p <* 0.001). With spm2d comparison of hip joint orientation, we detected no significant difference between cohorts. This finding may be the result of the small number of participants. However, our novel methods for analyzing hip motion in the natural environment are robust.

### 4.2. The Importance of Functional Calibration Procedures

ISB recommendations are used by researchers and clinicians to define anatomically relevant body-fixed axes and joint coordinate systems [60,61]. These recommendations were created (and adopted) to facilitate better communication by providing a set of standard, consistent definitions. While procedures for following ISB recommendations with camera-based motion-capture systems are well-developed, the same cannot be said for sensor-based systems. 

A functional calibration procedure is one that aligns sensor coordinate systems with anatomically relevant ones. These procedures are critical and must be performed to ensure sensor-derived results follow ISB recommendations (i.e., are anatomically relevant and comparable across different individuals and different data collections). Orientating sensor coordinate systems to anatomical relative ones helps reduce large errors and deviations from ISB recommendations that would result from misalignments between the sensor coordinate system (a result of initial sensor placement) and anatomically relevant body-fixed axes. Functional calibrations also correct for offsets. Our method, for example, corrects for offset by assuming neutral standing corresponds to 0° hip flexion/extension and adduction/abduction.

A camera-based motion capture system calibration trial uses a neutral standing posture. While IMU functional calibrations can be done with only static postures, this approach is usually not practical and measurements are also more precise when dynamic motions are used for calibration [4,10,11,15,30]. Our functional calibration procedure relied on the assumption that hip joint motion occurs predominantly in the sagittal plane during walking [11]. Fasel et al. applied this same assumption to hip motion during squats to determine the sagittal plane [10]. While an appropriate functional calibration ensures that sensor placement is not highly critical for accurate results [11], the effects of ill-defined body-fixed axes are still more evident in certain joint angle measures. Kinematic crosstalk (misinterpretation of a rotation about an axis as a rotation about another) is more evident with increasing flexion angles [62].

The implications and limitations of functional calibration procedures should be well understood. For example, our method assumed 0° of pelvic tilt during neutral standing (implied by 0° hip flexion/extension and adduction/abduction). Most individuals, however, assume a neutral standing position with 11–13° of anterior pelvic tilt [63,64] and pelvic tilt is thought to contribute to cam morphology [65]. Therefore, not accounting for differences in pelvic tilt during neutral standing had implications for our clinical research question. Because we assumed 0° of pelvic tilt during neutral standing, the spatial histograms were a better representation of excursions from neutral standing, not joint angles. Additionally, because individuals typically stand in slight hip flexion during neutral standing, our joint orientation results likely overrepresent maximum hip joint extension and underrepresent maximum flexion for all participants.

### 4.3. Magnetic Field Disturbances Reduce MIMU Accuracy 

Quaternions are determined by relying on gravity to define the global coordinate system’s vertical axis and a combination of angular velocity changes and the Earth’s magnetic field to define orientation in the plane perpendicular to gravity. Magnetic field data provide a heading relative to Earth’s magnetic north and angular velocity data provide correctional updates in the presence of magnetic field disturbances. If the magnetic field data are influenced by large magnetic field disturbances, the accuracy of quaternion estimation algorithms are decreased since a true heading relative to the Earth’s magnetic field cannot be accurately identified [66,67]. Depending on the degree of magnetic field disturbances in an environment, these inaccuracies may be a significant hurdle in data post-processing.

**Figure 7:**
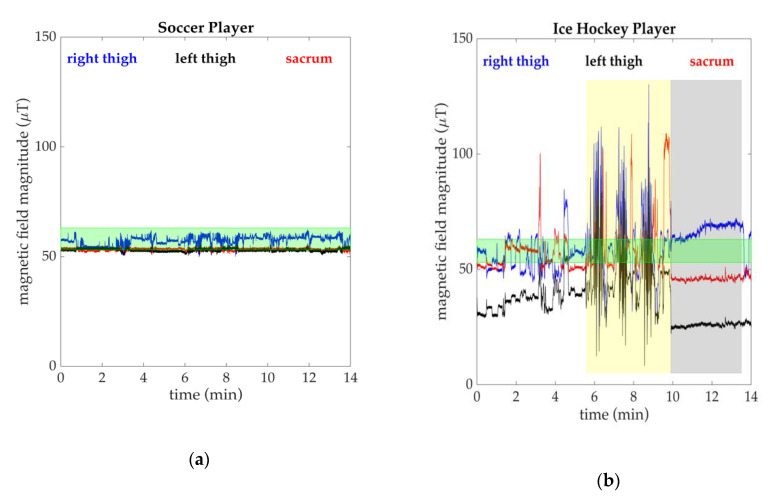
(**a**) Example of magnetic field magnitude recorded by MIMUs placed on a participant during a soccer practice. Deviations around 58 µT (the magnetic field in Boston) were within ±5 µT and kinematic calculations were therefore not affected by the magnetic field environment. The range of 53–63 µT is highlighted in green; (**b**) Example of magnetic field magnitude recorded by the MIMUs during an ice hockey practice. These deviations are large enough to affect kinematic calculations (extreme period highlighted in yellow) and magnetic field magnitudes were unequal and incorrect for the geographic location of data collection (highlighted in grey).

To further understand cam morphology, we chose to measure hip joint motion of at-risk soccer players despite ice hockey players being at even higher risk [68]. This choice was influenced by the added challenge of magnetic field disturbances that arise when using MIMUs to measure on an indoor ice hockey rink. We saw the effects of these disturbances in pilot data from ice hockey players. These data were highly influenced by large variations in the magnetic field in the data collection environments, likely caused by the refrigeration system under the indoor ice rink. In the geographic location where this research took place (Greater Boston Area, MA, USA), the magnitude of the Earth’s magnetic field is approximately 58 µT. The quaternion estimation algorithm developed by APDM Inc. can accurately account for magnetic field magnitude fluctuations of ±5 µT. Examples of magnetic field data over the course of 14 min at a soccer pitch and an ice hockey rink are shown in Figure 7. At the ice hockey rink (Figure 7b), the magnetic field magnitude fluctuated with deviations much greater than ±5 µT and the magnetic field magnitude was highly variable for the thigh and sacrum sensors. These large differences significantly decrease the accuracy of quaternion estimates, since differences in a sensor’s “understanding” of “ground truth” (Earth’s magnetic field) will affect the quaternion calculations. Magnetic field disturbances for all sensors during ice hockey practices were similar to the example shown, emphasizing the impact of the magnetic field on measurements taken in specific environments. Although we chose to exclude data that was impacted by magnetic field disturbances, correction algorithms for quaternion estimations have been implemented to address errors that arise from these disturbances [69,70,71]. Users who wish to not derive their own quaternions should use caution when using these estimations and verify that fluctuations in the magnetic field during data collections are within the acceptable range for the algorithm chosen. 

When using quaternions to derive joint angles, it is important to note that magnetic field disturbances may impact joint angle calculations differently—some measures are more sensitive to these disturbances than others. Magnetic field fluctuations have a greater impact on calculations of hip joint internal/external rotation than on those of flexion/extension and adduction/abduction when individuals are on their feet in upright positions [11]. In these positions, the majority of hip joint internal/external rotation lies in the plane perpendicular to gravity. Therefore, hip joint internal/external rotation calculations are highly sensitive to magnetic field disturbances.

### 4.4. Joint Angle Time Series Data 

**Figure 8:**
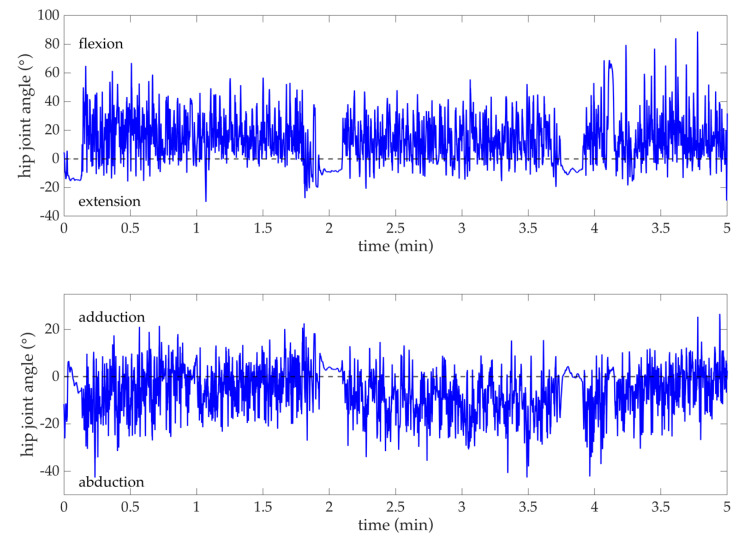
Example of one soccer player’s joint angle times series data ((**top**) flexion/extension, (**bottom**) adduction/abduction) from 5 min of practice. After coordinate system transformations (refer to Section 2.4) and functional calibration (refer to Section 2.5), hip joint angles were calculated using the orientation of thigh body fixed axes in the functionally calibrated sacrum sensor coordinate system (Equations (1)–(6)).

Hip joint flexion/extension and abduction/adduction (time series example shown in Figure 8) were calculated using the orientation of body fixed axes (Equations (1)–(6)). When using these equations with data transformed to the sacrum sensor coordinate system, these equations simplify since l_sacrum_ = [1 0 0], f_sacrum_ = [0 1 0] and t_sacrum_ = [0 0 1]. This simplification reveals that the orientations of two body fixed axes, l_thigh_ and t_thigh_, are particularly critical when calculating hip joint angles. To this effect, Horenstein et al. [11] reported that drift induced errors do not affect hip joint angle calculations equally and that these errors have the least impact on flexion/extension and the greatest impact on internal/external rotation joint angles. They concluded that internal/external rotation is likely the most susceptible to magnetic field disturbances since it describes joint rotation that (for the case of their study and this current study too) mostly lies in the plane perpendicular to gravity. Additionally, error propagation increases in calculations of flexion/extension (lowest) to internal/external rotation (highest) as the effects of an inaccurate orientation of t_thigh_ in the sacrum sensor coordinate system are “amplified.” 

### 4.5. Statics Periods May Be Susceptible to Errors

Because “ground truth” angular velocity is 0 rad/s during static periods, any gyroscope bias (angular velocity measurements offset from 0 rad/s when a sensor is stationary) leads to rapid accumulation of integration errors (integration of angular velocity to determine rotation relative to the starting position) [72]. During prolonged static periods, these accumulated errors can greatly affect IMU quaternion estimates since an accurate “ground truth” cannot be identified in the plane perpendicular to gravity. In data processing, these errors will present in results showing that a stationary sensor is slowly rotating. MIMUs are considered preferable to IMUs for longer duration recordings because they fuse angular velocity and magnetic field data for more accurate orientation estimates in the plane perpendicular to the direction of gravity. The Motion Studio fusion algorithm (developed by APDM Inc.) used in this study puts a variable weight on the magnetic field data depending on the surrounding disturbances. With increasing magnetic field disturbances there is a decreasing dependency on these data and an increasing dependency on angular velocity. We found that while the Motion Studio algorithm worked well for short duration magnetic field disturbances, prolonged static periods in combination with magnetic field disturbances were problematic. 

### 4.6. Future Work

Future work might focus on addressing the limitations of functional calibration procedures within the context of clinically relevant questions. Data could also be incorporated in machine learning algorithms to identify repetitive motion that are of clinical interest. While MIMUs cannot solely determine force magnitude, sensor-derived measures can provide insight into force direction. In the clinical context of understanding the etiology of cam morphology, current computational finite element models (which are commonly used to investigate the effect of mechanics on bone growth [73,74,75,76,77]) are limited by hypothetical magnitudes and directions of loading [75,78]. Future work in our group will address this limitation by incorporating sensor-derived hip joint orientation into computational models in efforts to better understand the etiology of cam morphology. Lastly, our methods could be further extended to find the joint orientations corresponding to the greatest motion intensities during activities.

## 5. Conclusions

We used MIMUs to measure adolescent hip motion of elite-level soccer players during sports practices and their recreationally active peers during afterschool activities. Although we did not detect a difference in hip joint orientation, we did find that athletes had a greater intensity level and variability in hip joint motion than controls. While wireless sensors can measure hip motion in a natural environment, there are still limitations that need be well-understood including large magnetic field disturbances, errors in angular velocity updates and functional calibrations that rely on assumptions (for example neutral standing defined by 0° flexion/extension and adduction/abduction). Despite these limitations, the work presented here provides a new method for analyzing hip joint motion using measures of intensity and 2d spatial orientation.

## Figures and Tables

**Table 1:** Participant demographics shown as mean ± standard deviation (SD).

Group	Number of Participants	Age (Years)	Organized Sport Participation (Hours/Week)
Athletes	*n* = 7	10.2 ± 1.28	8.46 ± 1.64
Controls	*n* = 7	9.63 ± 0.64	N/A

**Table 2:** Offsets (defined by the minimum baseline value after application of the average moving window) during activity and instructed neutral standing during the functional calibration. Differences in offsets between the athletes and controls were not found to be significant. Values are indicated as mean ± SD.

Group	Activity Offset (m/s^2^)	Instructed Neutral Standing Offset (m/s^2^)
athletes	1.63 ± 1.08	0.29 ± 0.090
controls	1.56 ± 1.32	0.15 ± 0.041

**Table 3:** Mean ± SD intensity level, intensity variability and time spent above the dynamic threshold of athletes during sports practices and controls during typical adolescent activities. Individual participant data are shown in Figure 5. Differences between the two groups were found to be statistically significant for all three variables (*p* < 0.001).

Group	Level (m/s^2^)	Variability (m/s^2^)	Time Above Dynamic Threshold (min)
athletes	7.57 ± 1.65	12.32 ± 2.08	69.76 ± 7.69
controls	3.62 ± 0.95	4.51 ± 2.26	16.85 ± 9.16

## Supplementary Materials

The following are available online at https://www.mdpi.com/1424-8220/20/17/4970/s1, Video S1: Visualizing Hip Joint Motion.
